# Adult choledochal cyst resection in Scandinavia: uncommon malignancy and considerable morbidity in a multicentre surgical cohort

**DOI:** 10.1093/bjsopen/zrag168

**Published:** 2026-11-03

**Authors:** Sondre Busund, Jennie Engstrand, Helena Taflin, Bobby Tingstedt, Linda Lundgren, Jozef Urdzik, Ioannis Gkekas, Ernesto Sparrelid, Sheraz Yaqub, Christian Sturesson

**Affiliations:** Department of HPB Surgery, Oslo University Hospital, Oslo, Norway; Institute of Clinical Medicine, University of Oslo, Oslo, Norway; Division of Surgery and Oncology, Department of Clinical Science, Intervention and Technology, Karolinska Institutet, Karolinska University Hospital, Stockholm, Sweden; Department of Transplantation, Sahlgrenska University Hospital, Gothenburg, Sweden; Institute of Clinical Sciences, Sahlgrenska Academy at University of Gothenburg, Gothenburg, Sweden; Department of Clinical Sciences Lund, Surgery, Lund University and Skåne University Hospital, Lund, Sweden; Department of Biomedical and Clinical Sciences, Linköping University, Linköping, Sweden; Clinical Department of Surgery in Linköping, Region Östergötland, Linköping, Sweden; Department of Surgical Sciences, Uppsala University, Uppsala, Sweden; Department of Diagnostics and Intervention, Surgery, Umeå University, Umeå, Sweden; Division of Surgery and Oncology, Department of Clinical Science, Intervention and Technology, Karolinska Institutet, Karolinska University Hospital, Stockholm, Sweden; Department of HPB Surgery, Oslo University Hospital, Oslo, Norway; Institute of Clinical Medicine, University of Oslo, Oslo, Norway; Division of Surgery and Oncology, Department of Clinical Science, Intervention and Technology, Karolinska Institutet, Karolinska University Hospital, Stockholm, Sweden

**Keywords:** biliary malformation, hepaticojejunostomy, Todani classification

Choledochal cysts are rare congenital dilatations of the biliary tree classified according to the Todani system^1^ (*Fig. S1*). With the increasing use of cross-sectional imaging, they are now more frequently identified in adults, often as incidental findings. The primary rationale for prophylactic resection is the presumed risk of malignant transformation, and complete cyst excision with Roux-en-Y hepaticojejunostomy is regarded as the standard treatment^2,3^. However, most evidence derives from Asian cohorts reporting malignancy rates of 7–11%^2^. In clinical practice in Northern Europe, malignant transformation appears to occur less commonly, but regional data remain limited. This study evaluated perioperative morbidity, long-term outcomes, and pathological findings after choledochal cyst resection in a Scandinavian cohort.

In this multicentre cohort study, adult patients undergoing surgery for choledochal cysts at seven Scandinavian hepatopancreatobiliary centres (six in Sweden, one in Norway) between 2010 and 2025 were identified. Patients with Todani type V disease were excluded. Given the small number of patients with malignancy, analyses were exploratory and hypothesis generating. Details of the methodology, complete results, and full data tables are provided in the *Supplementary material*.

Sixty-two patients were included (median age 47.3 years; 77% women). Todani type I cysts accounted for 73%, and type IV cysts for 24%. An anomalous pancreaticobiliary duct union was identified in 24% of patients (*Table 1*). Abdominal pain was the most common presenting symptom (66%), whereas 23% of patients were diagnosed incidentally (*Table S1*). Surgery was performed with prophylactic intent in 85% of patients. Cyst excision with Roux-en-Y hepaticojejunostomy was performed in 95% of patients, and an open approach was used in 79% of procedures (*Tables S2* and *S3*).

**Table 1:** Summary of cohort characteristics, outcomes, and pathology (*n* = 62)

**Demographics and presentation**	
Age at surgery (years), median (i.q.r.)	47.3 (29.6–57.7)
Sex	
Female	48 (77%)
Male	14 (23%)
Incidental finding	14 (23%)
Abdominal pain at presentation	41 (66%)
Todani type I	45 (73%)
Todani type IV	15 (24%)
APBDU present	15 (24%)
Common channel length* (mm), median (i.q.r.)	17 (14–23)
**Operative characteristics**	
Prophylactic intent	53 (85%)
Cyst excision + Roux-en-Y hepaticojejunostomy	59 (95%)
Open approach	49 (79%)
Minimally invasive approach	13 (21%)
**Short-term outcomes (≤ 90 days)**	
Major complication (Clavien–Dindo grade ≥ III)	20 (32%)
Bile leakage (ISGLS grade B/C)	12 (19%)
Postoperative pancreatic fistula (ISGPS grade B/C)	6 (10%)
**Long-term outcomes (> 90 days)**	
Biliary stricture	9 (15%)
Postoperative ERCP (≥ 1)	14 (23%)
Postoperative PTBD (≥ 1)	8 (13%)
Reoperation (> 90 days)	8 (13%)
Late readmission (> 90 days)	24 (39%)
Follow-up (months), median (i.q.r.)	70.4 (31.3–120.2)
**Pathology and malignancy**	
Extrahepatic bile duct pathology	
Normal	31 (50%)
Chronic inflammation	17 (27%)
Dysplasia (low/moderate)	8 (13%)
Invasive malignancy (any site)	4 (6%)

Values are *n* (%) unless otherwise stated. *Among those patients with APBDU. There was no significant difference in major complications between open or converted surgery and minimally invasive surgery (*P* = 0.740). Two patients in the open approach group were converted from minimally invasive approach. Full baseline, operative, complication, pathology, and follow-up data are provided in the *Supplementary material*. i.q.r., interquartile range; APBDU, anomalous pancreaticobiliary duct union; ISGLS, International Study Group of Liver Surgery; ISGPS, International Study Group of Pancreatic Surgery; ERCP, endoscopic retrograde cholangiopancreatography; PTBD, percutaneous transhepatic biliary drainage.

Major postoperative complications (Clavien–Dindo grade ≥ III) occurred in 32% of patients, including bile leakage in 19% and postoperative pancreatic fistula in 10%. Long-term morbidity remained substantial, with biliary strictures in 15% of patients and postoperative endoscopic retrograde cholangiopancreatography and percutaneous transhepatic biliary drainage required in 23% and 13% of patients, respectively. Reoperation beyond 90 days was required in 13% of patients, and 39% experienced at least one late readmission (*Table S4*).

Histopathology of the extrahepatic bile duct most commonly demonstrated normal findings (50%), chronic inflammatory changes (27%), or low- to moderate-grade dysplasia (13%). Invasive malignancy was identified in four patients (6%), comprising two gallbladder cancers, one extrahepatic cholangiocarcinoma, and one intrahepatic cholangiocarcinoma (*Table S5*). In three of these four patients, malignancy had already been suspected on preoperative imaging. The median follow-up was 70.4 months (*Table S6*).

In this Scandinavian multicentre surgical cohort, resection of choledochal cysts in adults was associated with substantial short- and long-term morbidity, whereas invasive malignancy was uncommon and, when present, was often suspected before surgery. Importantly, because only surgically treated patients were included, the observed frequency of malignancy should not be interpreted as the risk of malignant transformation among all adults with choledochal cysts.

A similar pattern of considerable morbidity and a low incidence of malignancy has been reported in a Western multi-institutional cohort^4^. Practice across Northern European centres varies considerably, particularly in borderline cases^5^. Although exploratory, these findings suggest that the contemporary burden of occult malignancy in Scandinavian adults with choledochal cysts may be lower than traditionally assumed, whereas the morbidity associated with prophylactic resection remains substantial. Improved risk stratification and prospective data, including non-operative cohorts, are needed to better inform treatment decisions.

## Supplementary Material

zrag168_Supplementary_Data

## Data Availability

Data are available upon request.

## References

[1] Todani T, Watanabe Y, Narusue M, Tabuchi K, Okajima K. Congenital bile duct cysts: classification, operative procedures, and review of thirty-seven cases including cancer arising from choledochal cyst. Am J Surg 1977;134:263–269 10.1016/0002-9610(77)90359-2 889044

[2] Ten Hove A, de Meijer VE, Hulscher JBF, de Kleine RHJ. Meta-analysis of risk of developing malignancy in congenital choledochal malformation. Br J Surg 2018;105:482–490 10.1002/bjs.10798 PMC5900735 29480528

[3] Bloomfield GC, Nigam A, Calvo IG, Dorris CS, Fishbein TM, Radkani P et al Characteristics and malignancy rates of adult patients diagnosed with choledochal cyst in the west: a systematic review. J Gastrointest Surg 2024;28:77–87 10.1016/j.gassur.2023.11.007 38353080

[4] Soares KC, Kim Y, Spolverato G, Maithel S, Bauer TW, Marques H et al Presentation and clinical outcomes of choledochal cysts in children and adults: a multi-institutional analysis. JAMA Surg 2015;150:577–584 10.1001/jamasurg.2015.0226 25923827

[5] Brudvik KW, Yaqub S, Kemsley E, Coolsen MME, Dejong CHC, Wigmore SJ et al Survey of the attitudes of hepatopancreatobiliary surgeons in Northern Europe to resection of choledochal cysts in asymptomatic Western adults. BJS Open 2019;3:785–792 10.1002/bjs5.50208 PMC6887667 31832585

