# Supplementary material for: Adult choledochal cyst resection in Scandinavia: uncommon malignancy and considerable morbidity in a multicentre surgical cohort
Source: BJS Open. 2026 Nov 3;10(6):zrag168. doi: 10.1093/bjsopen/zrag168 (PMC13729128; doi:10.1093/bjsopen/zrag168)
Supplement: Supplementary file 1 [file zrag168_supplementary_data.docx]

**Adult choledochal cyst resection in Scandinavia: uncommon malignancy and considerable morbidity in a multicentre surgical cohort**

Sondre Busund^1,2^, Jennie Engstrand^3^, Helena Taflin^4,5^, Bobby Tingstedt^6^, Linda Lundgren^7,8^, Jozef Urdzik^9^, Ioannis Gkekas^10^, Ernesto Sparrelid^3^, Sheraz Yaqub^1,2†^, Christian Sturesson^3†^

1. Department of HPB Surgery, Oslo University Hospital, Oslo, Norway
2. Institute of Clinical Medicine, University of Oslo, Norway
3. Division of Surgery and Oncology, Department of Clinical Science, Intervention and Technology, Karolinska Institutet, Karolinska University Hospital, Stockholm, Sweden.
4. Department of Transplantation, Sahlgrenska University Hospital, Gothenburg, Sweden.
5. Institute of Clinical Sciences, Sahlgrenska Academy at University of Gothenburg, Gothenburg, Sweden.
6. Department of Clinical Sciences Lund, Surgery. Lund University and Skåne University Hospital, Lund, Sweden.
7. Department of Biomedical and Clinical Sciences, Linköping University, Linköping, Sweden.
8. Clinical Department of Surgery in Linköping, Region Östergötland, Linköping, Sweden.
9. Department of Surgical Sciences, Uppsala University, Uppsala, Sweden.
10. Department of Diagnostics and Intervention, Surgery, Umeå University, Umeå, Sweden.

† Joint last authors

Corresponding author:

Christian Sturesson

Department of Surgery

Karolinska University Hospital

S-14186 Stockholm, Sweden

E-mail: christian.sturesson@ki.se

ORCID: https://orcid.org/0000-0003-3451-2840

**Supplementary Material - Index**

| **Supplementary Methods** | *page 3* |
| --- | --- |
| Study design and participating centres | *page 3* |
| Inclusion and exclusion criteria | *page 3* |
| Data collection | *page 3* |
| Definitions and outcomes | *page 3* |
| Statistical analysis | *page 3* |
| Ethical approval | *Page 4* |
| **Supplementary Results** | *page 4-5* |
| **Supplementary Discussion** | *page 5-6* |
| Strengths and limitations | *page 6* |
| **References** | *page 7* |
| **Supplementary Figures and Tables** | *page 8* |
| Fig. S1 Todani classification of choledochal cysts | *page 8* |
| Table S1 Baseline characteristics | *page 9* |
| Table S2 Operative characteristics | *page 10* |
| Table S3 Distribution across centres | *page 11* |
| Table S4 Short- and long-term complications | *page 12* |
| Table S5 Pathological findings | *page 13* |
| Table S6 Follow-up summary | *page 14* |
|  |  |

**Supplementary Methods**

**Study design and participating centres**

This retrospective multicentre cohort study included seven specialized hepatopancreatobiliary (HPB) centres (six in Sweden and one in Norway). Adult patients who underwent surgical treatment for choledochal cysts between January 2010 and December 2025 were identified through operative logs and institutional databases. Follow-up was defined as the time from surgery to the last clinical contact, death, or end of the study period. The administrative censoring date was set to 31 December 2025. Follow-up data were obtained through review of medical records and national registry data. Vital status was available for all included patients at the censoring date. No patient was lost to follow-up with respect to survival status. Outcome data were collected from the records of the participating centres. Surgical techniques and postoperative management followed the local practices of each institution. The study was reported in accordance with the STROBE statement for observational studies(1).

**Inclusion and exclusion criteria**

Adult patients (≥18 years) undergoing surgical treatment for choledochal cysts were included. Patients with Caroli disease (Todani type V) were excluded because of its distinct pathophysiology and management. Choledochal cyst was diagnosed from characteristic cystic or fusiform bile duct dilatation on cross-sectional imaging, classified by the Todani system(2) at each centre's multidisciplinary meeting, without a fixed diameter threshold. Imaging was not centrally re-reviewed.

**Data collection**

Electronic medical records were reviewed to extract demographic characteristics, clinical presentation, imaging findings, operative details, postoperative outcomes, and histopathological results. The presence of anomalous pancreaticobiliary duct union was recorded when documented on radiological imaging or during surgery. Indications for surgery were recorded as non-mutually exclusive categories, including symptoms, previous cyst-related complications, radiological suspicion of malignancy, and prophylactic resection. Prophylactic resection was defined as surgery performed in the absence of radiological suspicion of malignancy, regardless of symptom status.

**Definitions and outcomes**

Postoperative complications were classified according to the Clavien–Dindo classification(3). Bile leakage and postoperative pancreatic fistula were defined according to the International Study Group of Liver Surgery (ISGLS) and the International Study Group of Pancreatic Surgery (ISGPS) criteria, respectively(4, 5). Short-term outcomes were defined as events occurring within 90 days after surgery. Long-term outcomes included events occurring beyond 90 days, such as biliary strictures, biliary interventions, and late hospital readmissions. Biliary stricture was defined as radiological or endoscopic evidence of anastomotic narrowing associated with clinical symptoms, biochemical cholestasis, or the need for intervention. Histopathological findings were recorded as the most advanced lesion identified at each anatomical site. Dysplasia was categorized as low/moderate or high grade.

**Statistical analysis**

All data were entered into a REDCap database. Data quality checks were performed prior to analysis, which was conducted using R version 4.3.1 (R Foundation for Statistical Computing, Vienna, Austria). Continuous variables are presented as median (interquartile range), and categorical variables as number (percentage). Fisher's exact test was used to compare categorical data. Owing to the limited number of patients with malignancy, analyses were mainly exploratory and descriptive. Missing data were summarised for each variable and, when present, reported alongside denominators in all tables.

**Ethical approval**

This study was approved by the relevant regional ethics review board in Sweden (Approval No. 2024-06951-01) and Norway (Approval No. 939387). Written informed consent was obtained from all living patients in Norway. In Sweden, patient inclusion followed an opt-out procedure approved by the ethics review board. The requirement for informed consent was waived for deceased patients by the respective ethics committees.

**Supplementary Results**

Data not reported in the letter are highlighted here.

**Patient characteristics**

Patient demographics are summarised in Table S1. Among the 15 patients with an anomalous pancreaticobiliary duct union, the median common channel length was 17 mm. One patient (2%) reported a family history of cholangiocarcinoma. Endoscopic retrograde cholangiopancreatography (ERCP) was performed preoperatively in 19 patients (31%).

**Operative characteristics**

Operative details are shown in Table S2. The 62 patients were treated across seven centres over 15 years. Case volume and the use of minimally invasive surgery were unevenly distributed between centres and concentrated in the most recent period (Table S3). Two patients underwent reconstruction with hepaticoduodenostomy, and one patient underwent pancreatoduodenectomy. Concomitant liver resection was performed in five patients (8%). Biliary reconstruction involved a single anastomotic opening in 54 patients (87%), two openings in 6 (10%), and three openings in 2 (3%). The median duration of operation was 228 min, and estimated blood loss was low.

**Short-term outcomes**

Short-term outcomes are detailed in Table S4. Postoperative pancreatic fistula (ISGPS grade B/C) occurred in 6 patients (10%). All pancreatic fistulas occurred after standard cyst excision. The single patient undergoing pancreatoduodenectomy had no fistula. The median length of hospital stay was seven days. Readmission within 90 days occurred in 26% of patients. There was no significant difference in the rate of major postoperative complications between patients undergoing open or converted surgery (15 of 49) and those undergoing minimally invasive surgery (5 of 13) (P = 0.740). Bile leakage and biliary stricture were more frequent after reconstruction of more than one biliary opening than after a single opening (3 of 8 versus 9 of 53, P = 0.183; and 2 of 6 versus 7 of 52, P = 0.231, respectively).

**Long-term outcomes**

Long-term outcomes are detailed in Table S4. Biliary strictures, when present, typically developed within the first postoperative year (median 372 days). Reoperations after 90 days were performed for small bowel obstruction (4 patients), biliary stricture (1), incisional hernia (1), and other indications (3).

**Pathological findings**

Pathological findings are presented per organ in Table S5. No clear association was observed between Todani subtype or anomalous pancreaticobiliary duct union and pathological severity.

**Follow-up**

Follow-up details are shown in Table S6. Follow-up was calculated from the date of surgery until death or last clinical contact. The median follow-up duration was 70.4 months, with individual follow-up ranging from 8.5 to 180 months. At 1, 3, and 5 years after surgery, follow-up data were available for 95%, 73%, and 58% of patients, respectively.

**Supplementary Discussion**

Prophylactic cyst excision is widely recommended, largely on the basis of data from predominantly Asian cohorts(6). How far that recommendation extends to Western adults, who are increasingly identified incidentally, is uncertain. A survey of 30 hepatobiliary centres in Northern Europe demonstrated considerable variation in both the classification and management of choledochal cysts in asymptomatic adults(7). Recommendations for resection were primarily influenced by duct diameter and patient age. These findings suggest that, particularly in borderline cases, routine resection is not uniformly supported among Northern European HPB surgeons.

The rate of surgical resection, (that is, the number of resections divided by the population), for choledochal cysts in the participating centres, covering all of Sweden and a large part of Norway, representing a population of approximately 13 million, is higher than that reported in a nationwide study from the Netherlands(8). This finding may reflect a relatively proactive surgical approach to the management of choledochal cysts in Scandinavia during the study period.

In addition, the diagnosis of choledochal cyst is not always straightforward. This is particularly relevant for the fusiform subtype characterized by dilatation of the entire extrahepatic bile duct (Todani type Ic), as the common bile duct may dilate physiologically with increasing age and following cholecystectomy(9). In the present study, the median largest width of the extrahepatic bile duct was 21 mm. This overlap may complicate the distinction between congenital cystic dilatation and acquired biliary dilatation.

The observed malignancy rate of 6% in the present cohort is slightly lower than that reported in previous Western and Asian studies(10, 11). Both the previous and the present study derive malignancy rates from resected cohorts, which likely overestimate the risk in the broader populations of adults with choledochal cysts. Histopathological findings were predominantly characterized by chronic inflammatory changes and low- to moderate-grade dysplasia. This suggests that although epithelial alterations are common, progression to invasive cancer could be less frequent than has historically been assumed, at least in surgically treated Western adults.

Compared to the oncological yield, the procedural burden documented in the main article emphasizes that surgical treatment represents a major therapeutic commitment rather than a single prophylactic procedure. Thirteen patients (21%) underwent surgery using a minimally invasive surgery (MIS) approach. There was no significant difference in major complications between open or converted surgery and MIS (P = 0.740), but given the limited number of minimally invasive procedures, this comparison is underpowered and should be regarded as inconclusive rather than evidence of equivalence. In addition, MIS was unevenly distributed across the cohort. Ten of the thirteen patients operated with MIS approach underwent surgery in the most recent study period 2020–2025 and the procedures were concentrated to a few centres (Table S3). The small numbers and uneven distribution should be considered when interpreting the comparison of operative approach.

The prevalence of anomalous pancreaticobiliary duct union in the study cohort was markedly lower than that reported in Asian studies (12, 13), potentially reflecting differences in carcinogenic mechanisms or risk profiles. Given that malignancy in this cohort was frequently suspected preoperatively, these exploratory findings suggest that a more selective, risk-adapted approach, balancing oncological risk against the substantial morbidity of surgery, merits evaluation in larger prospective studies, rather than uniformly supporting resection for all adults with choledochal cysts.

**Strengths and limitations**

This study has several limitations. First, only surgically treated patients were included, so the cohort reflects referral patterns, selection practices, and treatment decisions at specialised centres rather than the full spectrum of adult choledochal cyst disease. The observed prevalence of malignancy, therefore, applies only to resected patients and should not be interpreted as the overall malignancy risk among all adults with choledochal cysts. Second, the retrospective design introduces a risk of selection bias and incomplete data capture. Variables with more than 10% missing data should be interpreted with caution and although survival follow-up was complete, long-term events managed outside the participating centres may have been missed. Third, diagnosis rested on cross-sectional imaging without a fixed diameter threshold and without central imaging review, potentially overlapping with age- or cholecystectomy-related duct dilatation. Fourth, the sample size was limited, with only four patients with malignancy the study is underpowered to identify predictors of malignancy or to perform adjusted analyses. Finally, surgical technique was not standardised across centres, and minimally invasive surgery was unevenly distributed, so comparisons of operative approach remain exploratory. Additional limitations include the lack of a non-operative control group, absence of centralized pathology review, and incomplete data on ethnicity and family history.

Despite these limitations, the study has important strengths, including its multicentre design, exclusive focus on adults, and detailed reporting of both perioperative and long-term outcomes.

1.	von Elm E, Altman DG, Egger M, Pocock SJ, Gøtzsche PC, Vandenbroucke JP. The Strengthening the Reporting of Observational Studies in Epidemiology (STROBE) statement: guidelines for reporting observational studies. J Clin Epidemiol. 2008;61(4):344–9.

2.	Todani T, Watanabe Y, Narusue M, Tabuchi K, Okajima K. Congenital bile duct cysts: Classification, operative procedures, and review of thirty-seven cases including cancer arising from choledochal cyst. The American Journal of Surgery. 1977;134(2):263–9.

3.	Dindo D, Demartines N, Clavien PA. Classification of surgical complications: a new proposal with evaluation in a cohort of 6336 patients and results of a survey. Ann Surg. 2004;240(2):205–13.

4.	Koch M, Garden OJ, Padbury R, Rahbari NN, Adam R, Capussotti L, et al. Bile leakage after hepatobiliary and pancreatic surgery: a definition and grading of severity by the International Study Group of Liver Surgery. Surgery. 2011;149(5):680–8.

5.	Bassi C, Marchegiani G, Dervenis C, Sarr M, Abu Hilal M, Adham M, et al. The 2016 update of the International Study Group (ISGPS) definition and grading of postoperative pancreatic fistula: 11 Years After. Surgery. 2017;161(3):584–91.

6.	Sastry AV, Abbadessa B, Wayne MG, Steele JG, Cooperman AM. What is the incidence of biliary carcinoma in choledochal cysts, when do they develop, and how should it affect management? World J Surg. 2015;39(2):487–92.

7.	Brudvik KW, Yaqub S, Kemsley E, Coolsen MME, Dejong CHC, Wigmore SJ, et al. Survey of the attitudes of hepatopancreatobiliary surgeons in northern Europe to resection of choledochal cysts in asymptomatic Western adults. BJS Open. 2019;3(6):785–92.

8.	de Kleine RH, Schreuder AM, Ten Hove A, Hulscher JBF, Borel Rinkes IHM, Dejong CHC, et al. Choledochal malformations in adults in the Netherlands: Results from a nationwide retrospective cohort study. Liver Int. 2020;40(10):2469–75.

9.	Beyer G, Kasprowicz F, Hannemann A, Aghdassi A, Thamm P, Volzke H, et al. Definition of age-dependent reference values for the diameter of the common bile duct and pancreatic duct on MRCP: a population-based, cross-sectional cohort study. Gut. 2023;72(9):1738–44.

10.	Ten Hove A, de Meijer VE, Hulscher JBF, de Kleine RHJ. Meta-analysis of risk of developing malignancy in congenital choledochal malformation. Br J Surg. 2018;105(5):482–90.

11.	Bloomfield GC, Nigam A, Calvo IG, Dorris CS, Fishbein TM, Radkani P, et al. Characteristics and malignancy rates of adult patients diagnosed with choledochal cyst in the West: a systematic review. J Gastrointest Surg. 2024;28(1):77–87.

12.	Kimura K, Ohto M, Ono T, Tsuchiya Y, Saisho H, Kawamura K, et al. Congenital cystic dilatation of the common bile duct: relationship to anomalous pancreaticobiliary ductal union. AJR Am J Roentgenol. 1977;128(4):571–7.

13.	Todani T, Watanabe Y, Fujii T, Uemura S. Anomalous arrangement of the pancreatobiliary ductal system in patients with a choledochal cyst. American Journal of Surgery. 1984;147(5):672–6.

**Supplementary Figures and Tables**

Fig. S1 Schematic illustration of the Todani classification of choledochal cysts

Type I, cystic dilatation of the extrahepatic bile duct; type II, saccular diverticulum of the extrahepatic bile duct; type III, choledochocele; type IVa, multiple cystic dilatations of both intrahepatic and extrahepatic bile ducts; type IVb, multiple cystic dilatations limited to the extrahepatic bile duct; type V, intrahepatic cystic dilatation (Caroli disease).

**Table S1** Baseline characteristics

| **Characteristic** | **Value** | **Missing** |
| --- | --- | --- |
| Patients, n | 62 |  |
| **Demographics and clinical status** | | |
| Age at surgery (years), median (i.q.r.) | 47.3 (29.6–57.7) |  |
| Women | 48 (77%) |  |
| Body mass index (kg/m²), median (i.q.r.) | 24.5 (21.9–27.4) | 6 (10%) |
| ASA ≥ III | 8 (13%) |  |
| ECOG ≥ 2 | 13 (21%) |  |
| **Presentation** | | |
| Incidental finding | 14 (23%) |  |
| Abdominal pain at presentation | 41 (66%) |  |
| Jaundice at presentation | 8 (13%) |  |
| **Anatomy and history** | | |
| Largest width of choledochal cyst (mm), median (i.q.r.) | 21 (15–35) |  |
| Length of choledochal cyst (mm), median (i.q.r.) | 40 (30–60) | 9 (15%) |
| APBDU present | 15 (24%) |  |
| Common channel length (mm), median (i.q.r.) (among APBDU present, n = 15) | 17 (14–23) | 1 (7%) |
| Previous abdominal surgery | 13 (21%) |  |
| Preoperative ERCP | 19 (31%) |  |
| Time from diagnosis to surgery (days), median (i.q.r.) | 305 (131–820) |  |
| Time from MDT conference to surgery (days), median (i.q.r.) | 93 (38–179) |  |
| **Todani classification** | | |
| Todani type I | 45 (73%) |  |
| Todani type II | 1 (2%) |  |
| Todani type III | 0 (0%) |  |
| Todani type IV (IVa + IVb) | 15 (24%) |  |
| Todani classification unknown | 1 (2%) |  |
| **Family history (self-reported)** | | |
| Family history (any: choledochal cysts/Caroli or primary liver/bile duct cancer) | 1 (2%) |  |
| Family history of choledochal cysts/Caroli | 0 (0%) |  |
| Family history of primary liver/bile duct cancer | 1 (2%) |  |
| No family history of the above | 52 (84%) |  |
| Family history unknown | 9 (15%) |  |

*Data are median (i.q.r.) or n (%). Missing data are shown only when present. APBDU, anomalous pancreaticobiliary duct union; ASA, American Society of Anesthesiologists; ECOG, Eastern Cooperative Oncology Group; ERCP, endoscopic retrograde cholangiopancreatography; i.q.r., interquartile range; MDT, multidisciplinary team.*

**Table S2** Operative characteristics

| **Characteristic** | **Value** | **Missing** |
| --- | --- | --- |
| Patients, n | 62 |  |
| **Indication for surgery (multiple answers may apply)** | | |
| Abdominal pain | 16 (26%) |  |
| Previous complications attributed to cyst | 8 (13%) |  |
| Suspicion of malignant transformation | 4 (6%) |  |
| Prophylactic surgery | 53 (85%) |  |
| Other indication | 1 (2%) |  |
| **Type of surgery** | | |
| Cyst excision + Roux-en-Y hepaticojejunostomy | 59 (95%) |  |
| Cyst excision + hepaticoduodenostomy | 2 (3%) |  |
| Pancreaticoduodenectomy | 1 (2%) |  |
| Concomitant liver resection | 5 (8%) |  |
| **Surgical approach** | | |
| Open | 47 (76%) |  |
| Laparoscopic | 2 (3%) |  |
| Robotic-assisted | 11 (18%) |  |
| Converted | 2 (3%) |  |
| **Number of separate biliary openings** | | |
| 1 | 54 (87%) |  |
| 2 | 6 (10%) |  |
| 3 | 2 (3%) |  |
| **Lymphadenectomy stations (multiple answers may apply)** | | |
| None | 35 (56%) |  |
| Station 8 (common hepatic artery) | 12 (19%) |  |
| Station 12 (hepatoduodenal ligament) | 21 (34%) |  |
| Station 13 (retropancreatic) | 1 (2%) |  |
| Station 16 (aorto-caval) | 1 (2%) |  |
| Duration of operation (min), median (i.q.r.) | 228 (176–348) | 6 (10%) |
| Estimated blood loss (ml), median (i.q.r.) | 50 (50–150) | 11 (18%) |

*Values are median (i.q.r.) for continuous variables and n (%) for categorical variables. Indications and lymphadenectomy stations are multi-select and multiple answers may apply. Missing data are shown only when present. i.q.r., interquartile range.*

**Table S3** Distribution of patients and minimally invasive approach across centres and study periods

| **Group** | **Patients, n** | **Minimally invasive, n** |
| --- | --- | --- |
| Total cohort | 62 | 13 (21%) |
| **By centre** |  |  |
| Karolinska | 26 | 4 |
| Oslo | 11 | 2 |
| Sahlgrenska | 9 | 6 |
| Uppsala | 7 | 0 |
| Lund | 5 | 0 |
| Linköping | 2 | 0 |
| Umeå | 2 | 1 |
| **By study period** |  |  |
| 2010–2014 | 16 | 2 |
| 2015–2019 | 19 | 1 |
| 2020–2025 | 27 | 10 |

*Minimally invasive denotes laparoscopic or robotic-assisted resection; the two converted procedures are counted as open.*

**Table S4** Short- and long-term complications

| **Characteristic** | **Value** | **Missing** |
| --- | --- | --- |
| Patients, n | 62 |  |
| **Short-term complications (≤ 90 days)** | | |
| Clavien–Dindo ≥ III | 20 (32%) |  |
| Grade III | 19 (31%) |  |
| Grade IV | 1 (2%) |  |
| 90-day mortality | 0 (0%) |  |
| Bile leakage (ISGLS grade B/C) | 12 (19%) | 1 (2%) |
| Pancreatic fistula (ISGPS grade B/C) | 6 (10%) | 1 (2%) |
| Length of hospital stay (days), median (i.q.r.) | 7 (5–11) |  |
| Readmission within 90 days | 16 (26%) | 1 (2%) |
| **Long-term complications (> 90 days)** | | |
| Postoperative biliary stricture (> 90 days) | 9 (15%) | 4 (6%) |
| Time from surgery to first stricture (days), median (i.q.r.) (among strictures with known dates) | 372 (100–710) |  |
| Postoperative ERCP ≥ 1 | 14 (23%) | 8 (13%) |
| Postoperative PTBD ≥ 1 | 8 (13%) | 8 (13%) |
| Reoperation after > 90 days | 8 (13%) | 1 (2%) |
| Readmission to hospital after > 90 days | 24 (39%) | 15 (24%) |
| **Cause of reoperation(s) (> 90 days) (multiple answers may apply)** | | |
| Small bowel obstruction | 4 (6%) |  |
| Biliary stricture | 1 (2%) |  |
| Gastrointestinal bleeding | 0 (0%) |  |
| Incisional hernia | 1 (2%) |  |
| Other | 3 (5%) |  |

*Data are median (i.q.r.) or n (%); missing shown only when present. ERCP, endoscopic retrograde cholangiopancreatography; i.q.r., interquartile range; ISGLS, International Study Group of Liver Surgery; ISGPS, International Study Group of Pancreatic Surgery; PTBD, percutaneous transhepatic biliary drainage. ERCP, PTBD, reoperation and readmission after > 90 days are patient-level indicators (≥ 1 event). Time to first stricture is calculated among strictures with known dates. Causes of reoperation may include more than one indication per patient.*

**Table S5** Pathological findings

| **Characteristic** | **Value** |
| --- | --- |
| Patients, n | 62 |
| Total malignancies (any site) | 4 (6%) |
| **Gallbladder pathology (most severe finding)** | |
| Previously resected | 18 (29%) |
| **available, n = 44** |  |
| Normal | 11 (25%) |
| Chronic cholecystitis | 22 (50%) |
| Dysplasia (low/moderate) | 3 (7%) |
| Dysplasia (high grade / Tis) | 1 (2%) |
| Cancer (adenocarcinoma) | 1 (2%) |
| Other benign | 5 (11%) |
| Other malignant | 1 (2%) |
| **Extrahepatic bile duct pathology (most severe finding) - available, n = 62** | |
| Normal | 31 (50%) |
| Chronic inflammation | 17 (27%) |
| Dysplasia (low/moderate) | 8 (13%) |
| Dysplasia (high grade) | 1 (2%) |
| Cancer (adenocarcinoma) | 1 (2%) |
| Other benign | 4 (6%) |
| **Liver pathology (if liver resection performed) - resected, n = 5** | |
| Liver resection performed | 5 (8%) |
| Normal | 2 (40%) |
| Dysplasia (low/moderate) | 0 (0%) |
| Dysplasia (high grade) | 0 (0%) |
| Cancer (adenocarcinoma) | 1 (20%) |
| Other benign | 2 (40%) |

*Total malignancies represent patients with malignancy in any assessed specimen. Gallbladder percentages are calculated among available gallbladders (excluding previously resected). Tis, carcinoma in situ.*

**Table S6** Follow-up summary

| **Characteristic** | **Value** |
| --- | --- |
| Patients, n | 62 |
| Time origin | Date of surgery |
| Administrative censor date | 31 December 2025 |
| Follow-up (months), median (i.q.r.) | 70.4 (31.3–120.2) |
| Follow-up (months), range | 8.5–180.3 |
| Total follow-up time (person-years) | 396.6 |
| Under observation at 1 year | 59 of 62 (95%) |
| Under observation at 3 years | 45 of 62 (73%) |
| Under observation at 5 years | 36 of 62 (58%) |

*Time origin was defined as date of surgery. Follow-up was calculated from surgery to death or last known date alive, administratively censored at 31 December 2025. Values are median (i.q.r.) for continuous variables and n (%) for categorical variables. i.q.r., interquartile range.*
